# Dual intron-targeted CRISPR-Cas9-mediated disruption of the AML *RUNX1-RUNX1T1* fusion gene effectively inhibits proliferation and decreases tumor volume in vitro and in vivo

**DOI:** 10.1038/s41375-023-01950-9

**Published:** 2023-07-18

**Authors:** Signe Neldeborg, Johannes Frasez Soerensen, Charlotte Thornild Møller, Marie Bill, Zongliang Gao, Rasmus O. Bak, Kasper Holm, Boe Sorensen, Mette Nyegaard, Yonglun Luo, Peter Hokland, Magnus Stougaard, Maja Ludvigsen, Christian Kanstrup Holm

**Affiliations:** 1 Department of Pathology, Aarhus University Hospital, Aarhus, Denmark; 2 Department of Clinical Medicine, Aarhus University, Aarhus, Denmark; 3 Department of Hematology, Aarhus University Hospital, Aarhus, Denmark; 4 Department of Biomedicine, Aarhus University, Aarhus, Denmark; 5 Department of Clinical Biochemistry, Aarhus University Hospital, Aarhus, Denmark; 6 Department of Health Science and Technology, Aalborg University, Aalborg, Denmark; 7 Steno Diabetes Center Aarhus, Aarhus University Hospital, Aarhus, Denmark; 8 Danish Life Science Cluster, Copenhagen, Denmark

**Keywords:** Translational research, Acute myeloid leukaemia

## Abstract

Oncogenic fusion drivers are common in hematological cancers and are thus relevant targets of future CRISPR-Cas9-based treatment strategies. However, breakpoint-location variation in patients pose a challenge to traditional breakpoint-targeting CRISPR-Cas9-mediated disruption strategies. Here we present a new dual intron-targeting CRISPR-Cas9 treatment strategy, for targeting t(8;21) found in 5–10% of de novo acute myeloid leukemia (AML), which efficiently disrupts fusion genes without prior identification of breakpoint location. We show in vitro growth rate and proliferation reduction by 69 and 94% in AML t(8;21) Kasumi-1 cells, following dual intron-targeted disruption of *RUNX1-RUNX1T1* compared to a non t(8;21) AML control. Furthermore, mice injected with *RUNX1-RUNX1T1*-disrupted Kasumi-1 cells had in vivo tumor growth reduction by 69 and 91% compared to controls. Demonstrating the feasibility of *RUNX1-RUNX1T1* disruption, these findings were substantiated in isolated primary cells from a patient diagnosed with AML t(8;21). In conclusion, we demonstrate proof-of-principle of a dual intron-targeting CRISPR-Cas9 treatment strategy in AML t(8;21) without need for precise knowledge of the breakpoint location.

## Introduction

Acute myeloid leukemia (AML) is characterized by proliferation of undifferentiated myeloid cells. A sizeable fraction of AML cases is characterized by recurrent cytogenetic aberrations as well as genetic mutations affecting genes involved in the hematopoiesis. Among these, core-binding factor (CBF) AMLs are characterized by balanced translocations affecting the transcription factor CBF subunits and are divided into two subtypes; inv(16)(p13.1q22)/t(16;16)(p13.1;q22) and t(8;21)(q22;q22.1) (AML t(8;21)), resulting in the fusion genes *CBFB-MYH11* and *RUNX1-RUNX1T1* [1].

These subtypes, accounting for an estimated 15% of de novo AML cases, are associated with a favorable prognosis compared with other AML subtypes with an estimated 5-year overall survival of 60–70% [2–4]. While first-line treatment consisting of high-dose anthracycline and cytarabine with the possibility of adding gemtuzumab ozogamicin, will usually result in more than 90% of patients achieving a complete remission [5, 6], there is an unmet for novel strategies, as an estimated 30% of patients will experience a relapse [7, 8]. Disease monitoring is essential for identifying primary treatment response and early relapse, which is accomplished by determining measurable residual disease (MRD) primarily by monitoring the disease-defining translocations in peripheral blood and bone marrow [9]. Furthermore, as recent advances in the treatment of AML have primarily benefitted the younger patient population [10], there is a collective need for novel treatment strategies with low inherent toxicity that can target MRD in order to prevent relapse and perhaps act as bridging to allogeneic hematopoietic stem cells transplantation. Just as important, such a venture might allow for more efficient cytoreduction in the elderly patients without compromising safety [11].

In animal models, *RUNX1-RUNX1T1* has been shown to be independently insufficient to instigate AML t(8;21) leukemogenesis, and it is believed that additional genetic aberrations are necessary in order to cause AML [12]. However, as *RUNX1-RUNX1T1* has been shown to both inhibit the differentiation of the hematopoietic cells as well as aid in evasion of apoptotic cell death, the fusion gene is hypothesized to constitute a key factor in the maintenance of the leukemic cell population [13–15], making *RUNX1-RUNX1T1* an attractive putative target for medical intervention [16].

Novel therapies for AML are continuously being introduced to the treatment regimens for AML patients, with Bcl-2, *FLT3* and *IDH1/2* inhibitors representing the new wave of available therapies. In addition, progress is being made in CAR-T cell based treatment modalities. However, a major challenge yet to be overcome is identifying AML specific antigens that prevent CAR-T mediated damage of healthy cells [17]. In spite of these recent treatment advances, AML remains a malignancy with few available treatment options and a high overall mortality [18].

Gene editing using the clustered regularly interspaced palindromic repeats (CRISPR)-Cas9 offers the possibility to target pre-defined DNA sequences in human cells [19–21]. Recent advances in the CRISPR-Cas9 technique have demonstrated both feasibility and efficaciousness in treatment of transfusion-dependent β-thalassemia, sickle cell disease, aromatic L-amino acid decarboxylase (AADC) deficiency and transthyretin amyloidosis [22–26], demonstrating the potential power of CRISPR-Cas9 methodologies in precision medicine.

We hypothesize that a novel dual intron-targeting CRISPR-Cas9 methodology [27] can be used to disrupt *RUNX1-RUNX1T1* without damaging wild type *RUNX1* and *RUNX1T1*. By targeting two meticulously selected intron regions, flanking the fusion breakpoint, it is possible to induce major CRISPR-Cas9-mediated gene-disrupting deletions, without knowing the precise location of the breakpoint. Double strand break repair by non-homologous end-joining (NHEJ) joins together exons with out-of-frame codons following CRISPR-Cas9 cleavage causing a shift in the fusion gene reading frame resulting in a premature stop codon and thereby non-functional oncogene. This dual intron-targeting approach negates the need for precise knowledge of the fusion breakpoint location in patients offering a gene therapy solution without a need for preceding fusion breakpoint sequencing. The only genetic information needed to qualify for treatment would be the standard diagnostic t(8;21) identification, which can be available less than 72 h after the diagnosis is made.

In this proof-of-principle study, we demonstrate that the *RUNX1-RUNX1T1* can be targeted and disrupted utilizing a dual intron-targeting CRISPR-Cas9-mediated strategy elucidating this novel methodology’s potential in future treatment of AML t(8;21) patients.

## Methods

### Cell lines and patient samples

The human CBF AML cell line Kasumi-1 (ATCC, Manassas, VA, USA), positive for t(8;21)(q22;q22.1) was used for *RUNX1-RUNX1T1* disruption experiments, with AML cell line THP-1 (ATCC), which does not harbor the t(8;21) translocation, as negative control and the immortalized human dermal fibroblast cell line, MJ26146 as non-malignant control. Cells were cultured in RPMI-1640 Medium, with L-glutamine and sodium bicarbonate (MERCK, Burlington, MA, USA) supplemented with 20% fetal Bovine Serum (FBS) (GIBCO, Thermo Fisher Scientific, Waltham, MA, USA) and 1% Penicillin-Streptomycin (GIBCO) at 37 °C and 5% CO_2_. Archival mononuclear cells, preserved in DMSO in liquid nitrogen from a peripheral blood sample or bone marrow sample collected as part of routine diagnostic workup from four patients diagnosed with CBF AML with t(8;21)(q22;q22.1) at the Department of Hematology at Aarhus University Hospital, Denmark were utilized (Supplementary Table 1). Informed consent and ethical approval were waived due to the anonymous and proof-of-concept nature of this study (the Central Denmark Region Committees on Health Research Ethics, reference: 186/2017 and the Danish Data Protection Agency, reference: 727067/1-16-02-173-21).

### CRISPR-Cas9 gene editing

**Fig. 1:**
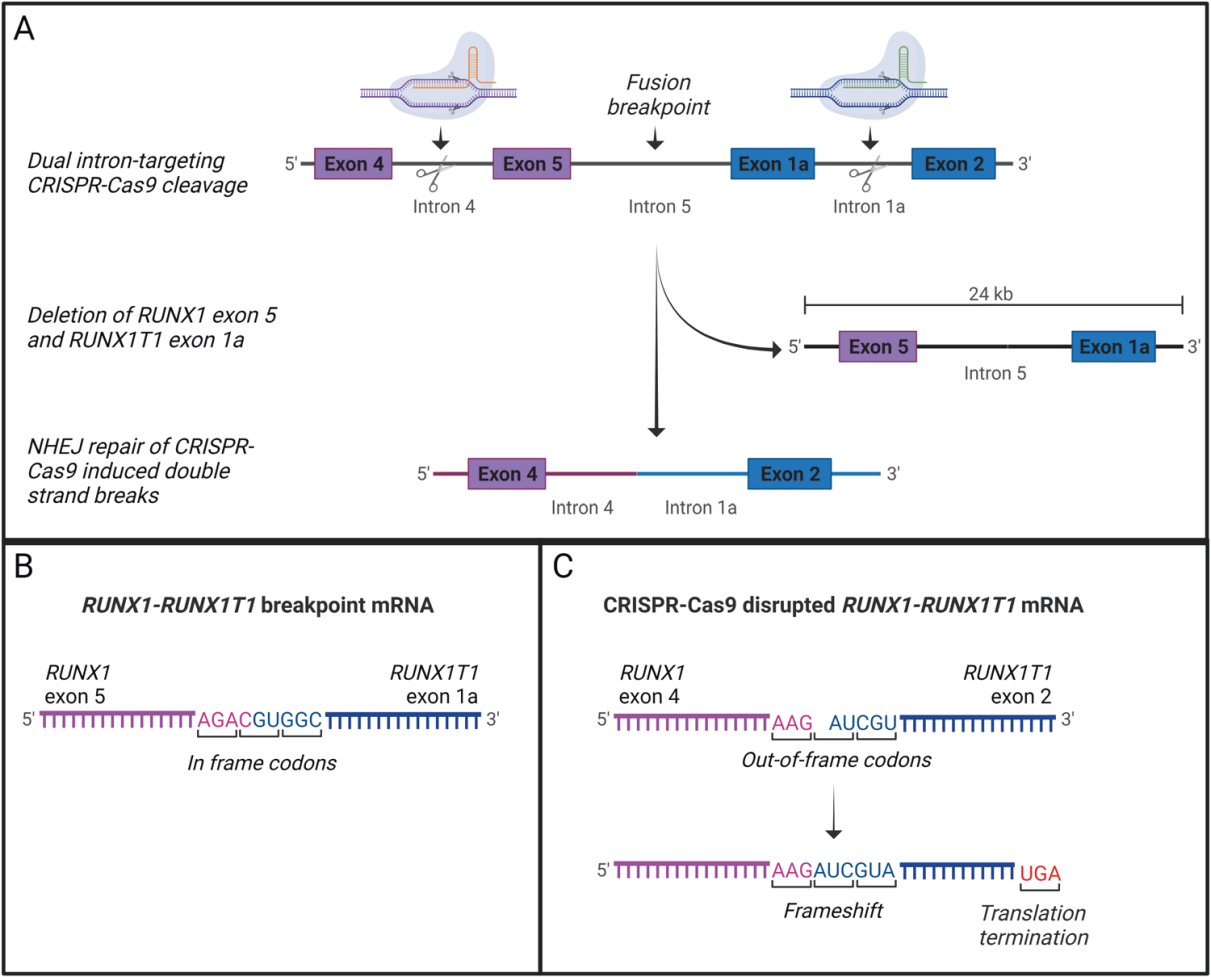
Fusion-gene disruption strategy. **A** Dual intron-targeting CRISPR-Cas9-mediated disruption of *RUNX1-RUNX1T1*. sgRNAs targeting intron 4 of *RUNX1* (orange) and intron 1a of *RUNX1T1* (green) are indicated. Cas9 cleavage at target sites within intron 4 of *RUNX1* and intron 1a of *RUNX1T1* leads to a major deletion of exon 5 in *RUNX1* and exon 1a of *RUNX1T1*. Through NHEJ repair introns 4 and 2 of *RUNX1* and *RUNX1T1*, respectively, are joined together. The product is a disrupted *RUNX1-RUNX1T1* fusion gene with a major deletion resulting in loss of the essential Runt domain and a shift in the reading frame. **B** The *RUNX1-RUNX1T1* fusion-gene mRNA showing the fusion of *RUNX1* exon 5 and *RUNX1T1* exon 1a. Both exons have in-frame codons and the fusion results in a functional oncogene. **C** The CRISPR-Cas9-disrupted *RUNX1-RUNX1T1* fusion-gene mRNA showing the fusion of *RUNX1* exon 4 and *RUNX1T1* exon 2. *RUNX1T1* exon 2 has out-of-frame codons, that when fused to the in-frame codons of *RUNX1* exon 4 causes a shift in the reading frame resulting in a premature stop codon and thereby non-functional oncogene.

Synthetic guide RNA molecules (sgRNAs) were designed to target intron regions in the *RUNX1-RUNX1T1* fusion gene (Synthego, Menlo Park, CA, USA) (Supplementary Table 2 and Fig. 1). Two sgRNAs were targeted against the *RUNX1* intron region between exon 4 and 5 and further two sgRNAs were targeted against the intron region between exon 1a and 2 in *RUNX1T1*. The sgRNA target sites did not contain common single nucleotide polymorphisms ensuring robust target sites with minimal potential patient-to-patient Cas9 cleavage efficiency variation. Target sites were also placed in intron regions without functions in splicing or other regulatory mechanisms. Ribonucleoprotein (RNP) complexes comprising sgRNAs and Streptococcus pyogenes Cas9 (spCas9) (Integrated DNA Technologies, Coralville, IA, USA) in a 2.6:1 ratio were preassembled on ice. Kasumi-1 and THP-1 cells were adjusted to desired concentration in Opti-MEM (Thermo Fisher Scientific) prior to transfection. Transfection was performed in 25 µl reactions in electroporation strip tubes (Lonza Basel, Switzerland) by electroporation (CM138, 4D-Nucleofector^TM^ X, Lonza). Controls treated with Cas9 without sgRNAs were included. The same CRISPR-Cas9 approach was used to target the *RUNX1-RUNX1T1* in mononuclear cells from the patient. The CRISPR-Cas9-mediated disruption of the *RUNX1-RUNX1T1* fusion gene was validated using targeted PCR and Sanger sequencing (Mix2Seq, Eurofins, Luxembourg).

The NGS based off-target analysis was performed using a custom ampliseq panel designed to cover the target sites for the RX2 and RXT1 guides (on-target) as well as the top 48 and 42 off-target sites, respectively, predicted by CRISPRoff (v1.2beta) software [28–30]. The panel was designed for standard DNA (275 bp) to maximize the coverage of the top off-target sites for each guide resulting in coverage of the two on-target sites as well as 90 off target sites. The libraries were made using the AmpliSeq™ Library PLUS for Illumina with AmpliSeq™ UD Indexes for Illumina® according to manufacturer’s protocol. The quality of the libraries was checked using a bioanalyzer and the concentration determined using qubit and sequenced using an NextSeq 500 with the NextSeq 500/550 High Output Kit v2.5 (300 Cycles). The bioinformatic off-target analysis was performed using BWA MEM for alignment and crispRvariants for mutation efficiency calculations [31].

### Polymerase chain reaction

DNA was purified from cells using the QIAamp DNA Mini Kit (QIAGEN, Hilden, Germany) according to manufacturer’s instructions. Ten to 100 nanograms of input DNA and primers targeting intron regions surrounding sgRNAs in *RUNX1* and *RUNX1T1* (MERCK) (Supplementary Table 2) were added to a 20 µl PCR reaction with DreamTaq DNA Polymerase and buffer (Thermo Fisher Scientific) according to manufacturer’s instructions. Targets were amplified on a thermal cycler as following: (1) Initial denaturation at 95 °C for 3 min, (2) 40 cycles of denaturation at 95 °C for 30 s, (3) annealing at 58 °C for 30 s, (4) extension at 72 °C for 1 min, (5) final extension at 72 °C for 7 min. The PCR products were visualized by capillary electrophoresis using the QIAxcel Advanced System (QIAGEN) and agarose gel electrophoresis. QX DNA Alignment Marker 15 bp/1 kb (QIAGEN) was used for PCR product size estimation.

### Cell growth monitoring

Following sgRNAs:Cas9 treatment, Kasumi-1 and THP-1 cells were transferred to 48 well-plates in triplicates and cultured in RPMI-1640 (MERCK) supplemented with 20% FBS (GIBCO) and 1% Penicillin-Streptomycin (GIBCO) at 37 °C, 5% CO_2_. On experiment days 2, 4, 6, 8, 10 and 12 cells were taken out, mixed with Solution 18, AO•DAP (Chemometec, Allerod, Denmark) and counted in NC slide A8 (Chemometec) using the NucleoCounter NC-250 (Chemometec).

### Cell proliferation

Following sgRNAs:Cas9 treatment, Kasumi-1 and THP-1 cells were split into triplicates and recovered for 24 h before staining with CellTrace Violet Cell (CTV) Proliferation Kit (7.5 µM, Invitrogen) according to manufacturer’s protocol. On experiment days 2, 4, 6, 9 and 11, 50,000 cells were stained with Zombie NIR viability dye (BioLegend, San Diego, CA, USA) before flow cytometry analysis (NovoCyte 3000RYB with 13 detectors (ACEA Biosciences Inc., San Diego, CA, USA. Agilent, Santa Clara, CA, USA)). Theoretically, the signal intensity will half by each proliferative cycle. Median fluorescence intensity (MFi) values were compared for treatment/control samples to determine the change in proliferation following treatment. Data was acquired using NovoExpress version 1.5 (ACEA Biosciences, Inc.) and analyzed using FlowJo version 10.7.1 (BD, Ashland, OR, USA).

### Cell sorting

Following sgRNAs:Cas9 treatment, Kasumi-1 cells were recovered for 24 h before staining with CTV (7.5 µM, Invitrogen, Thermo Fisher Scientific) according to manufacturer’s protocol. On day 10 after staining, 8 µg/ml propidium iodide (PI, BD Bioscience, San Jose, CA, USA) was added to the cell suspension. Immediately after, cells were sorted on a FACSAria III equipped with four lasers, 405, 488, 561 and 633 nm (BD Bioscience). The definition of CTV high and CTV low subpopulations were based on peak MFi values (Supplementary Figs. 1–4). Area scaling was 0.6, a 100 µm nozzle and 20 psi was used. Cells were sorted and collected at 4 °C. Data were collected using FACSDiva software version 8.0.2 (BD Bioscience).

### RT-qPCR

*RUNX1-RUNX1T1* gene expression was measured in triplicates in purified RNA from Kasumi-1 cells, THP-1 cells, and cells from peripheral blood and bone marrow of two patients diagnosed with AML t(8;21), respectively. The assay was performed in accordance with current clinical guidelines as previously described [32, 33].

### Animal studies

Six BALB/cAnNRj-Foxn1 nu/nu mice were injected with CRISPR-Cas9 *RUNX1-RUNX1T1*-disrupted Kasumi-1 cells or Cas9-control-treated Kasumi-1 cells in the right and left flank, respectively. Animals were sacrificed 4 weeks after injection and tumor volume was evaluated using the caliper method and the formula; *V* = (*W*^2^ × *L*)/2.

### Statistics

All estimates are reported with a 95% confidence interval (CI) and *p* values < 0.05 were considered statistically significant. Two-means comparisons for continuous variables were performed using Student’s *t* test or the Wilcoxon–Mann–Whitney test depending the distribution of the data. Multiple-means comparisons for continuous variables were performed using a two-way ANOVA with Geisser-Greenhouse correction. Prism version 8.2.1 (GraphPad Software, Inc. San Diego, CA, USA) was used for statistical analysis as well as design of figures. Regression for analysis of covariance was conducted using Stata version 15.1 (StataCorp LLC, College Station, TX, USA).

## Results

### Dual intron-targeting CRISPR-Cas9 disruption of the oncogenic driver *RUNX1-RUNX1T1* leads to effective inhibition of AML t(8;21) cancer cell growth and proliferation in vitro

**Fig. 2:**
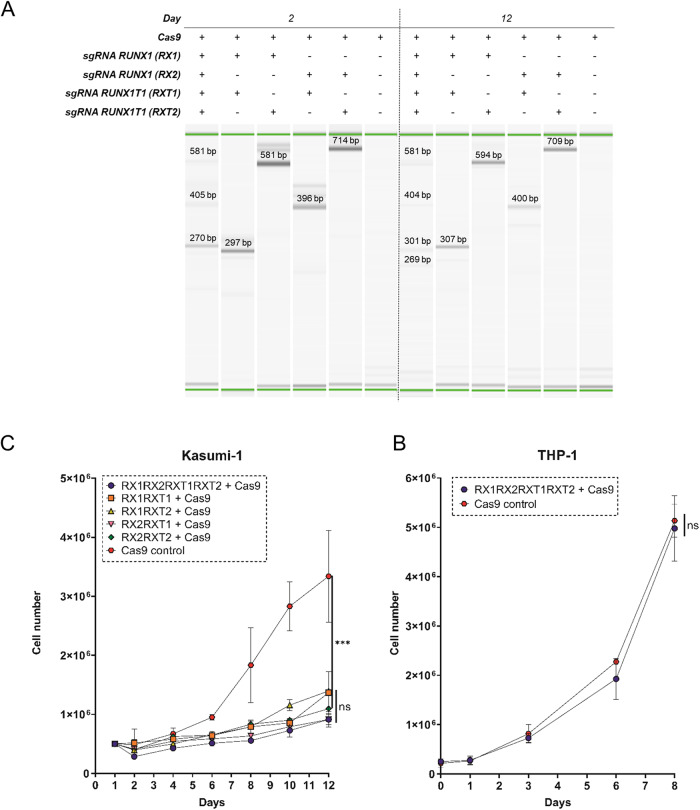
In vitro *RUNX1-RUNX1T1* disruption effectively inhibits AML *t(8;21)* cancer cell growth. **A** PCR analysis of CRISPR-Cas9 cleavage products from sgRNAs targeting intron 4 of *RUNX1* (RX1 and RX2) and intron 1a of *RUNX1T1* (RXT1 and RTX2) on day 2 and 12 following electroporation delivery of RNPs to Kasumi-1 cells. Theoretical PCR product sizes (bp) RX1-RX2-RXT1RXT2: 296, 391, 577 and 672; RX1-RXT1: 296; RX1-RXT2: 577; RX2-RXT1: 391; RX2-RXT2: 672; no guides: 24,416. Green lines at 15 and 1000 bp: capillary electrophoresis alignment marker. Bands close above and below theoretical band sizes are on-target CRISPR-Cas9 cleavage products with DNA repair generated indels. **B** Cell population growth in Kasumi-1 cells 12 days following electroporation delivery of RNPs to Kasumi-1 cells. sgRNAs were combined in five different configurations; RX1-RXT1, RX1-RXT2, RX2-RXT1, RX2-RXT2 and RX1-RX2-RXT1-RXT2. ***p = 0.0001, ns *p* > 0.05. **C** Cell population growth in THP-1 cells 8 days following electroporation delivery of RNPs to THP-1 cells. sgRNAs used were RX1-RX2-RXT1-RXT2. ns *p* = 0.934.

**Fig. 3:**
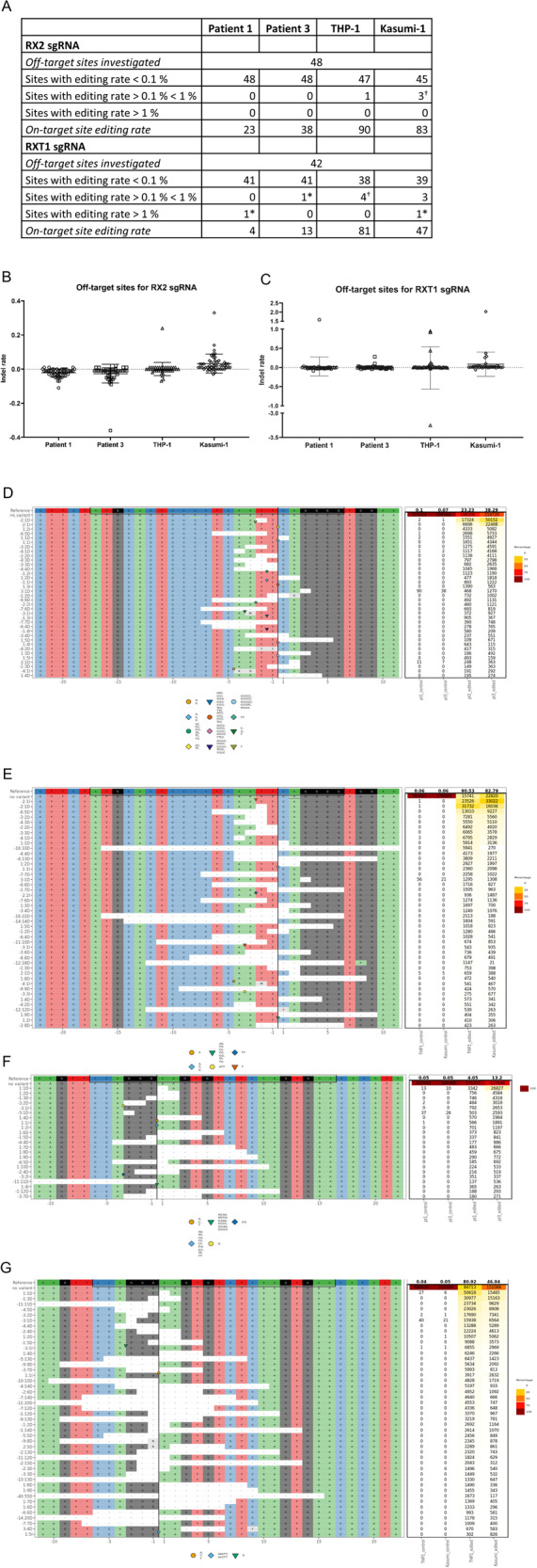
Off-target effects and on-target editing frequencies in AML t(8;21) patients, Kasumi-1, and THP-1 cell lines following CRISPR-Cas9 mediated *RUNX1-RUNX1T1* disruption. **A** Sequencing results for 48 and 42 top off-target sites for RX2 and RNXT1, respectively, predicted using CRISPRoff software. *high editing rate is due to a high indel count both in the control and edited sample for the given position due to the presence of polymorphisms. Therefore, we do not consider this an off-target effect. ^†^One is as above described*. **B** Indel frequencies for 48 off-target sites for RX2 sgRNA in Patient 1, Patient 3, Kasumi-1 cells, and THP-1 cells. **C** Indel frequencies for 42 off-target sites for RXT1 sgRNA in Patient 1, Patient 3, Kasumi-1 cells, and THP-1 cells. **D** Most frequent on-target indels for RX2 in Patient 1 and 3. The black box encompasses the RX2 gRNA and PAM sequence. The vertical black line indicates the theoretical cleavage site of Sp-Cas9 at PAM + 3. **E** Most frequent on-target indels for RX2 in Kasumi-1 and THP-1. The black box encompasses the RX2 gRNA and PAM sequence. The vertical black line indicates the theoretical cleavage site of Sp-Cas9 at PAM + 3. **F** Most frequent on-target indels for RXT1 in Patient 1 and 3. The black box encompasses the RX2 gRNA and PAM sequence. The vertical black line indicates the theoretical cleavage site of Sp-Cas9 at PAM + 3. **G** Most frequent on-target indels for RXT1 in Kasumi-1 and THP-1. The black box encompasses the RX2 gRNA and PAM sequence. The vertical black line indicates the theoretical cleavage site of Sp-Cas9 at PAM + 3.

We hypothesized that a *RUNX1-RUNX1T1* disruption in the AML t(8;21) cell line, Kasumi-1, would lead to reduced proliferation and cell population growth. Four sgRNAs were designed to target intron regions at various sites flanking the *RUNX1-RUNX1T1* breakpoint in pairs. By using this dual-guide CRISPR-Cas9-mediated approach in Kasumi-1, we were able to induce a major deletion in *RUNX1-RUNX1T1* involving exons following exon 4 of *RUNX1* and exons preceding exon 2 of *RUNX1T1*. By this, the function of the fusion gene was disrupted both by generation of frame shift in *RUNX1T1* and through deletion of functional domains in *RUNX1* (Fig. 1 and Supplementary Fig. 5). Different combinations of the four guides were tested and all sgRNA pairs were able to produce *RUNX1-RUNX1T1*-disruption through major deletions (Fig. 2A). Potential off-target effects were examined by targeted sequencing of 48 and 42 possible off-target sites for RX2 and RXT1 sgRNAs, respectively. No major off-target events were observed (Fig. 3A–C and Supplementary Figs. 6–9). On-target efficiencies for RX2 sgRNA were 23%, 38%, 90%, and 83% and for RXT1 sgRNA were 4%, 13%, 81%, and 47%, for Patient 1, Patient 3, THP-1, ad Kasumi-1, respectively (Fig. 3).

To investigate whether the disruptions affected Kasumi-1 cell population growth, the number of cells was determined at different time points through 12 days following the CRISPR-Cas9-mediated *RUNX1-RUNX1T1* disruption for all sgRNA combinations. All sgRNA pairs caused a significant reduction in cell population growth in Kasumi-1 by a median of 69.4% (range 61.0–74.5%) (*p* < 0.0002 (range 0.0002–<0.0001)) (Fig. 2B). As expected, no difference in growth was observed in the THP-1 control cell line (Fig. 2C) as well as no significant difference was observed between the reduced population growth among the different sgRNA pairs in Kasumi-1 cells (Fig. 2B). Due to similar performance on *RUNX1-RUNX1T1* disruption and cell-population growth reduction in Kasumi-1, sgRNA pair (RX2-RXT1) was used for all for further investigations.

**Fig. 4:**
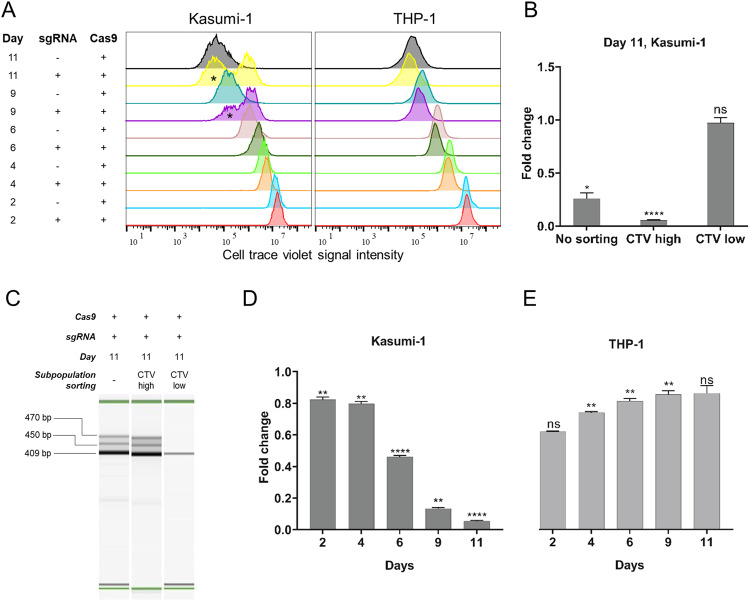
In vitro *RUNX1-RUNX1T1* disruption effectively inhibits AML t(8;21) cancer cell proliferation. **A** Flow cytometry analysis of cell proliferation rate 2, 4, 6, 9 and 11 days following electroporation delivery of RNPs (RX2-RXT1) to Kasumi-1 (left) and THP-1 cells (right). Asterisk (*) marks the in silico sorted CTV-low subpopulations at day 9 and 11 which were excluded from the analysis. **B** Fold change Kasumi-1 cell proliferation rate in the in silico sorted CTV high and CTV low subpopulations 11 days following electroporation delivery of RNPs (RX2-RXT1). CTV cell trace violet signal intensity. No sorting (left) *p = 0.011, CTV high (middle) ****p < 0.0001, CTV low (right) nsp = 0.993. **C** PCR analysis of CRISPR-Cas9 cleavage products 11 days following electroporation delivery of RNPs (RX2-RXT1) to Kasumi-1 and flow cytometry CTV-signal-intensity based in vitro sorting. No sorting: day 11 cell population before sorting, CTV high: day 11 cell population with high CTV signal intensity, CTV low: day 11 cell population with low CTV signal intensity. Theoretical PCR product sizes (bp) for RX2-RXT1 = 391. Green lines at 15 and 1000 bp: capillary electrophoresis alignment marker. Bands above theoretical band size are on-target CRISPR-Cas9 cleavage products with DNA repair generated insertions. **D** Fold change Kasumi-1 cell proliferation rate 2, 4, 6, 9 and 11 days following electroporation delivery of RNPs (RX2-RXT1). D2 **p = 0.002, D4 **p = 0.001, D6 ****p < 0.0001, D9 **p = 0.004, D11 ****p < 0.0001. **E** Fold change THP-1 cell proliferation rate 2, 4, 6, 9 and 11 days following electroporation delivery of RNPs (RX2-RXT1). D2 ns *p* = 0.056, D4 **p = 0.001, D6 **p = 0.002, D9 **p = 0.003, D11 ns *p* = 0.054.

To investigate whether the observed reduced cell growth was due to a reduced proliferation rate in the *RUNX1-RUNX1T1*-disrupted Kasumi-1 cells, the proliferation was monitored, day 2 through day 11. Proliferation was observed as a reduction in CTV dye intensity over time. Two subpopulations in the Kasumi-1 population treated with RX2-RXT1 were observed on day 9 and 11 (Fig. 4A). In silico sorting of the subpopulations on day 11, showed that the CTV low subpopulation had a 2.8% (*p* = 0.993) decreased proliferation rate and the other CTV high subpopulation a 94.6% (*p* < 0.0001) decreased proliferation rate compared to the non-disrupted Kasumi-1 cells control (Fig. 4B).

Flow cytometry mediated cell sorting on basis of CTV signal intensity followed by PCR showed that the highly proliferative subpopulation (CTV low) had a smaller proportion of cells with effectively disrupted *RUNX1-RUNX1T1* compared to the less proliferative subpopulation (CTV high), explaining the difference in proliferative potential within the RX2-RXT1 treated Kasumi-1 cells (Fig. 4C). Overall, we observed a gradual reduction in proliferation rate over the culture period following the *RUNX1-RUNX1T1* disruption in the Kasumi-1 cells (Fig. 4D). THP-1 cells treated with the *RUNX1-RUNX1T1-*disrupting sgRNAs RX2-RXT1 showed a non-significant 29% increase in proliferation rate on day 11, compared to untreated THP-1 cells (Fig. 4E) showing that dual intron-targeting CRISPR-Cas9 *RUNX1-RUNX1T1* disruption only affects Kasumi-1, cells harboring *RUNX1-RUNX1T1*, and not THP-1 cells which do not have the targeted fusion gene. Together, these results show that dual intron-targeting CRISPR-Cas9-mediated disruption of the oncogenic driver *RUNX1-RUNX1T1* leads to a profound inhibition of AML t(8;21) cancer cell growth and proliferation in vitro.

### Dual intron-targeting of *RUNX1-RUNX1T1* can induce a gene fusion in cell lines not carrying the fusion gene

We detected trace amounts of the disrupted *RUNX1-RUNX1T1* with a similar *RUNX1-RUNX1T1* sequence in THP-1 to the one induced in Kasumi-1 following treatment with RX2-RXT1 (Supplementary Fig. 10a) indicating, as expected, a CRISPR-Cas9 cleavage activity in both *RUNX1* and *RUNX1T1* introns in THP-1 cells. We monitored the THP-1 cells for a detectable cleavage product via gel electrophoresis for 6 days following treatment. The translocation continued to be detectable after 6 days (Supplementary Fig. 11). However, given that we detected no functional (i.e., non-disrupted) *RUNX1-RUNX1T1* sequence in the RX2-RXT1-treated THP-1 cells, we infer from these data that the presence of the disrupted *RUNX1-RUNX1T1* sequence could be due to an event caused by the CRISPR-Cas9-mediated disruption of *RUNX1* and *RUNX1T1* in THP-1 followed by faulty NHEJ repair, commonly characterizing cancer cell lines. This was confirmed by sequencing showing a similar break point to the one induced in Kasumi-1 following treatment with RX2-RXT1 (Supplementary Fig. 10b). We performed RT-qPCR on the RX2-RXT1-treated THP-1 cells and did not detect any *RUNX1-RUNX1T1* mRNA (data not shown). In order to evaluate the risk of generating this gene fusions in healthy cells, we then investigated RX2-RXT1 mediated disruption in a fibroblast cell line, MJ26146. We did indeed observe the presence of a translocation between chromosomes 8 and 21 corresponding to the disrupted *RUNX1-RUNX1T1* fusion. The fusion continued to be detectable after 10 days of observation (Supplementary Fig. 11). These data suggest that induction of translocations in healthy cells when using dual sgRNAs targeting introns is a legitimate risk when using CRISPR-Cas9, and should be investigated further in future research.

### *RUNX1-RUNX1T1* disruption and expression downregulation is feasible in primary AML cells from t(8;21) positive patients

**Fig. 5:**
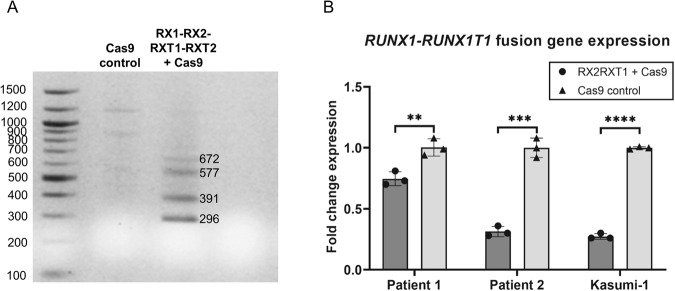
CRISPR-Cas9- mediated *RUNX1-RUNX1T1* disruption and mRNA expression downregulation in AML t(8;21) patient cells. **A** PCR analysis of CRISPR-Cas9 cleavage products from AML t(8;21) patient 4 cells following electroporation of RNPs (RX1-RX2-RXT1-RXT2). Theoretical PCR product sizes (bp) RX1-RX2-RXT1-RXT2 = 296, 391, 577 and 672; no guides: 24,416. **B** *RUNX1-RUNX1T1* fusion gene mRNA expression in a bone marrow sample (patient 1) and peripheral blood sample (patient 2) from two AML t(8;21) patients. The *RUNX1-RUNX1T1* mRNA levels were measured by RT-qPCR. Bars indicate mean of three technical replicates with error bars indicating s.e.m. Patient 1 **p = 0.0091, Patient 2 ***p = 0.0009, Kasumi-1 ****p < 0.0001.

Next, we wanted to evaluate whether the disruption observed in the cell model applies to patient AML cells. To this end, we performed CRISPR-Cas9-mediated *RUNX1-RUNX1T1* disruption utilizing our in vitro setup with the four sgRNAs (RX1, RX2, RXT1, RXT2) and showed the specific target site variations in a pre-therapeutic blood sample from one patient (Patient 4) diagnosed with CBF AML with t(8;21)(q22;q22.1). The *RUNX1-RUNX1T1* disruption was confirmed with PCR (Fig. 5A) and Sanger sequencing (Supplementary Fig. 12). The remaining three patients (Patient 1, 2, and 3) were *RUNX1-RUNX1T1* disrupted using only RX2 and RXT1. We showed significant downregulation of *RUNX1-RUNX1T1* expression following CRISPR editing in two of the patients (Fig. 5B) thus providing evidence that the CRISPR-Cas9-mediated method for fusion gene disruption is feasible also in patient-derived AML t(8;21) cancer cells. These data thus suggest that this new approach is also feasible for targeting primary human cancer cells.

### *RUNX1-RUNX1T1* disruption decreases tumor volume in vivo

**Fig. 6:**
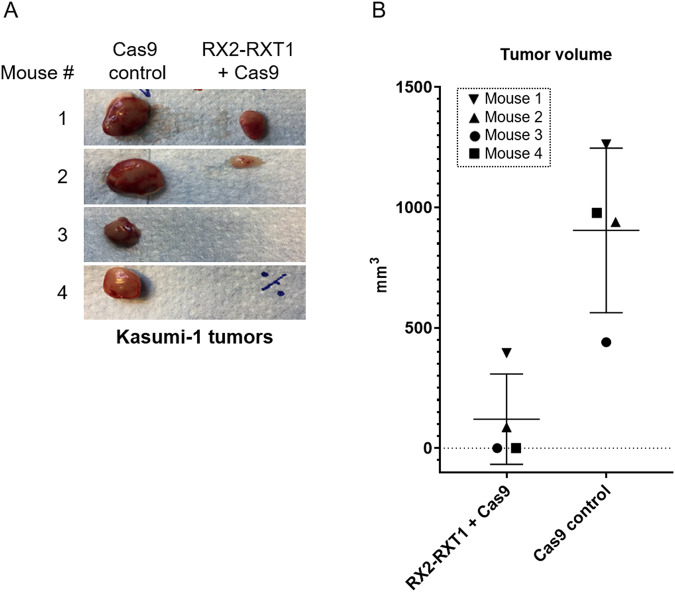
*RUNX1-RUNX1T1* disruption decreases tumor volume in vivo. **A** In vivo formed Kasumi-1 tumors removed from left (Cas9 control) and right (RX2-RXT1 + Cas9) flank of the four out of six treated mice that developed tumors 4 weeks following tumor cell injection. **B** Estimated tumor volume (mm^3^) for the four mice that developed tumor.

Lastly, we wanted to evaluate whether the Kasumi-1 population growth reduction following *RUNX1-RUNX1T1* disruption would also translate to reduced tumor growth in vivo. *RUNX1-RUNX1T1*-disrupted Kasumi-1 cells were injected into the right flanks of six immunodeficient nude mice. Each mouse also received an injection of non-disrupted Kasumi-1 cells into the corresponding left flank as control. Over 4 weeks, two out of six mice developed tumors in both flanks. Further two mice developed tumors, but only in the non-disrupted control flank. The last two mice did not develop tumors in either flank (Fig. 6A). In the two mice with tumor development in both flanks, tumor volumes were decreased by 69 and 91% in the *RUNX1-RUNX1T1*-disrupted flank, as compared to the control flank (Fig. 6B). These results support our in vitro findings and show that disruption of *RUNX1-RUNX1T1* disruption leads to a decrease in tumor volume also in vivo.

## Discussion

There is a collective need for novel treatment strategies with low inherent toxicity that can target MRD in AML t(8;21) in order to prevent relapse, offer efficient cytoreduction in the elderly patients without compromising safety and perhaps act as bridging to allogeneic hematopoietic stem cells transplantation.

In this proof-of-principle study, we have demonstrated that dual intron-targeting CRISPR-Cas9-mediated disruption of *RUNX1-RUNX1T1* leads to a reduction of chimeric *RUNX1-RUNX1T1* fusion transcripts and a significant decrease in AML t(8;21) leukemic tumor cell proliferation and growth both in vitro and in vivo. We have shown the feasibility of using standardized sgRNAs to disrupt the *RUNX1-RUNX1T1* in the Kasumi-1 cell line, in both an in vitro setting, to reduce tumor population size and proliferation, and in an in vivo mouse model where *RUNX1-RUNX1T1* disruption led to a tumor-volume reduction. We also proved able to induce disruption of *RUNX1-RUNX1T1* in AML t(8;21) patient-derived cells showing the feasibility of CRISPR-Cas9-mediated gene editing of primary leukemic cells. While the literature has described a second hit as necessary for development of leukemia in the presence of *RUNX1-RUNX1T1* [12, 34–39], our findings confirm the reported *RUNX1-RUNX1T1* dependence of AML t(8;21) cells for sustaining the leukemic cell population [13–15, 40]. Collectively, our data suggests that dual intron-targeting CRISPR-Cas9-mediated *RUNX1-RUNX1T1* disruption represents a potential new future therapy modality for AML patients with t(8;21)(q22;q22.1), a gene therapy solution without a need for preceding fusion breakpoint sequencing as the only genetic information needed to qualify for treatment would be the standard diagnostic t(8;21) identification.

As described by Martinez-Lage et al. [27], a feasible CRISPR-Cas9-mediated genome editing strategy to specifically disrupt fusion gene driven cancers should ensure that (1) the sgRNAs are designed to target only intron regions, avoiding disruption of exon regions and thereby affect expression of wild type alleles and (2) that the sgRNA target sites encompasses all patient specific breakpoints and fusion gene isoforms. We adhered to these criteria by locating our sgRNAs in intron regions, without single nucleotide polymorphisms, that encompass most known clinically relevant breakpoints in AML t(8;21). Furthermore, the location of our target sites ensured a disruption of the essential *RUNX1* Runt domain, responsible for DNA binding and protein-protein interactions of both the native *RUNX1* and the fusion protein *RUNX1-RUNX1T1*, thus not relying solely on frameshift causing deletions to disrupt *RUNX1-RUNX1T1* activity, making the disruption strategy more robust. Initially, multiple sgRNAs were used to target the *RUNX1-RUNX1T1* fusion gene in leukemic cells in vitro and in vivo and showed equal efficacy. Given the individual efficacy of all sgRNAs used in this study, we expect this approach to offer robust targeting of the fusion gene as well the potential variations in individual patient sequences, thus offering the possibility of a “one size fits all” approach to future gene therapy of AML t(8;21).

In the flow cytometry-based cell-proliferation study, we observed two subpopulations in Kasumi-1 cells treated with sgRNAs RX2-RXT1. Sorting of the two subpopulations on basis of proliferative signal followed by a PCR specific for the CRISPR-Cas9-disrupted *RUNX1-RUNX1T1* indicated that the two subpopulations were a result of imperfect delivery or low efficiency of ribonucleoprotein (RNP) complexes. One subpopulation had a lower fraction of disrupted *RUNX1-RUNX1T1* as compared to the other and showed similar proliferative potential as the Kasumi-1 cells treated without sgRNAs (Fig. 4C). At least two factors could explain this observation, first, the efficiency of the individual sgRNA in inducing deletions at the target site would indeed affect how large a fraction of the Kasumi-1 cells could be expected to harbor a disrupted *RUNX1-RUNX1T1* fusion gene following electroporation. A second factor is the efficiency of the electroporation-based delivery utilized in this study which could theoretically be amended by developing a more efficacious method for delivery of CRISPR-Cas9 to the target cells. We speculate that both factors contribute to the two subpopulations observed in our study when analyzing with flow cytometry. Future research efforts should be allocated toward quantification of the efficiency of individual sgRNA as well as optimizing delivery of CRISPR-Cas9 to leukemic cells.

Even though delivery of CRISPR-Cas9 components was previously achieved through electroporation by Frangoul et al. [22] in a transplant setting, this approach is not likely to be feasible in the setting of AML treatment as transplantations are allogeneic and not autologous. This clinical challenge necessitates further investigation of in vivo delivery of CRISPR-Cas9-mediated treatments in a clinical setting. An alternative to electroporation-based delivery when utilized as patient treatment could be an adeno-associated virus (AAV) derived vector with high affinity for the hematopoietic cells, such as the AAV serotype 6 [24, 41]. Generally, AAVs have been shown to have a high transfection efficiency which makes them candidates for clinical CRISPR-Cas9 delivery [26, 42]. A challenge with AAVs could arise if patients require multiple administrations, as adaptive mechanisms of the patients immune system, such as neutralizing antibodies or T-cells, can hinder multiple treatment cycles [43]. However, work is being done to engineer AAVs to evade immune responses [44] to overcome this particular challenge. Another delivery strategy, lipid nanoparticles, have been shown to instigate a low immune response potentially allowing for repeated treatment cycles with CRISPR-Cas9 [25, 45]. Speculatively, based on the clinical efficacy of anti-CD33 targeting in AML t(8;21) using gemtuzumab ozagamicin [46], a vector carrying the CRISPR-Cas9 payload, e.g. an AAV or a lipid nanoparticle, targeted toward CD33+ cells could be pursued in order to ensure high fidelity toward leukemic cells while potentially sparing healthy cells. Future research should aim to clarify the potential of different delivery approaches and their transduction efficacy in AML. Ultimately, the potential success of CRISPR-Cas9-mediated fusion gene disruption as a treatment for AML will depend on the ability to deliver the technological components to the leukemic cells efficiently in vivo.

We detected an unexpected effect of Cas9-cleavage in the THP-1 cell line leading to the formation of a t(8;21) translocation identical to the CRISPR-Cas9-disrupted *RUNX1-RUNX1T1* fusion in the Kasumi-1 cell line (Supplementary Fig. 10). However, no fusion transcript mRNA (data not shown) or growth inhibition could be detected in the in vitro studies (Fig. 2C) and only a non-significant increase in proliferation was observed in the cell population harboring a subpopulation with the disrupted *RUNX1-RUNX1T1* translocation (Fig. 4E). The findings were confirmed in a fibroblast cell line demonstrating that the observed fusion was not due to an inherent deficiency of the DNA repair pathway in the leukemic cell line. We suggest that the disrupted fusion gene in THP-1 cells was a product of error prone DNA repair mechanisms such as NHEJ and Microhomology-mediated end joining (MMEJ) which have been shown to be upregulated and implicated in both primary and secondary development of fusion-driven leukemias increasing the risk of chromosomal translocations following CRISPR-Cas9 induced double strand breaks [47–49]. However, nothing in our data suggested the formation of a neo-oncogene in the THP-1 cells as the gene fusion is similar to the disrupted (i.e., non-functional) *RUNX1-RUNX1T1* gene fusion created in the Kasumi-1 cells following treatment with CRISPR-Cas9 (Supplementary Fig. 10b) [50]. Our results clearly indicate that the technology is associated with the risk of generating gene fusions in the non-targeted cells. As such, future research should carefully evaluate the implications of these fusions in murine models.

In summary, the dual intron-targeting CRISPR-Cas9 technology can effectively inhibit proliferation and decrease tumor volume in AML t(8;21). However, potential side effects include induction of translocations whose potential effect on healthy cells need to be rigorously investigated preclinically. While the current data are intriguing, future studies focusing on strategies for in vivo delivery of the technology, benchmarking the efficacy in combination with currently approved treatments along with off-target effects studies will be necessary for this technology to reach AML t(8;21) patients.

## Supplementary information

Supplementary information

## Data Availability

The datasets generated and analyzed during the current study are available from the corresponding authors on reasonable request.
