# Supplementary material for: Dual intron-targeted CRISPR-Cas9-mediated disruption of the AML RUNX1-RUNX1T1 fusion gene effectively inhibits proliferation and decreases tumor volume in vitro and in vivo
Source: Leukemia. 2023 Jul 18;37(9):1792–801. doi: 10.1038/s41375-023-01950-9 (PMC10457201; doi:10.1038/s41375-023-01950-9)
Supplement: Supplementary file 1 — Supplementary information [file 41375_2023_1950_MOESM1_ESM.docx]

**Supplementary information**

Neldeborg and Soerensen *et al*., Dual intron-targeted CRISPR-Cas9-mediated disruption of the AML *RUNX1-RUNX1T1* fusion gene effectively inhibits proliferation and decreases tumor volume *in vitro* and *in vivo*

**Supplementary Table 1**

Patient characteristics

| Patient ID | Age at diagnosis | Sex | Year of diagnosis | Blast (%) | Blast immunophenotype | Karyotype |
| --- | --- | --- | --- | --- | --- | --- |
| 1 | 28 | M | 2016 | 50% (BM) | CD45lowCD34+CD117+HLA-DR+CD13+CD38+CLEC12A+CD7+CD19+ | 46,XY,t(8;21)(q22;q22) [20]/46,XY [5] |
| 2 | 56 | F | 2017 | 50% (PB) | CD45lowCD34+CD117+HLA-DR+CD13+CD38+CD33-CLEC12A+CD56+CD19(+) | 46,XX,t(8;21)(q22;q22) [23]/46,XX [2] |
| 3 | 79 | F | 2011 | 35% (PB) | CD45lowCD34+CD117lowHLA-DR+CD13+CD33+CLEC12A+CD56+CD64(+) | 46,XX,t(8;21)(q22;q22) [25] |
| 4 | 34 | F | 2013 | 65% (PB) | CD45lowCD34+CD117+HLA-DR+CD13+CD33-CLEC12A+ | 46,XX,t(8;21)(q22;q22) [25] |

*BM, bone marrow; F, female; M, Male; PB, peripheral blood.*

**Supplementary Table 2**

sgRNA and primer sequences

| **gRNA** | **Sequence 5' - 3'** |
| --- | --- |
| *RUNX1* sgRNA 1 (RX1) | AUUCCUGGUCAAGAUCAGCU |
| *RUNX1* sgRNA 2 (RX2) | AUGCACUCCCCUCAAUUCAG |
| *RUNX1T1* sgRNA 1 (RXT1) | GUUCACUUGAGACACUUCCC |
| *RUNX1T1* sgRNA 2 (RXT2) | UUGCUUGCUAAAGAUCUAUA |
| **Primer** | **Sequence 5’ - 3’** |
| *RUNX1* F | CTTTAGGTCATGCTTTTCAGAG |
| *RUNX1* R | CTTTGATACCTCCTACTCATCGC |
| *RUNX1T1* F | CTGTCACTCAAGGAATGTTGAC |
| *RUNX1T1* R | CCTTCCATATTTCCAGACAATG |

**Supplementary Figure 1**

Gating strategy for *RUNX1-RUNX1T1* disrupted Kasumi-1 cell populations and Cas9 controls to obtain a measure of the cell trace violet (CTV) median fluorescence intensity (MFi) from live Kasumi-1 cells on days 2, 4, 6, 9 and 11 following CRISPR-Cas9 treatment compared to controls. Here, gating is shown for Kasumi-1 cells 6 days following CRISPR-Cas9 treatment. The same strategy was used on data from day 2, 4, 9 and 11. Zombie NIR was used as viability marker.

**
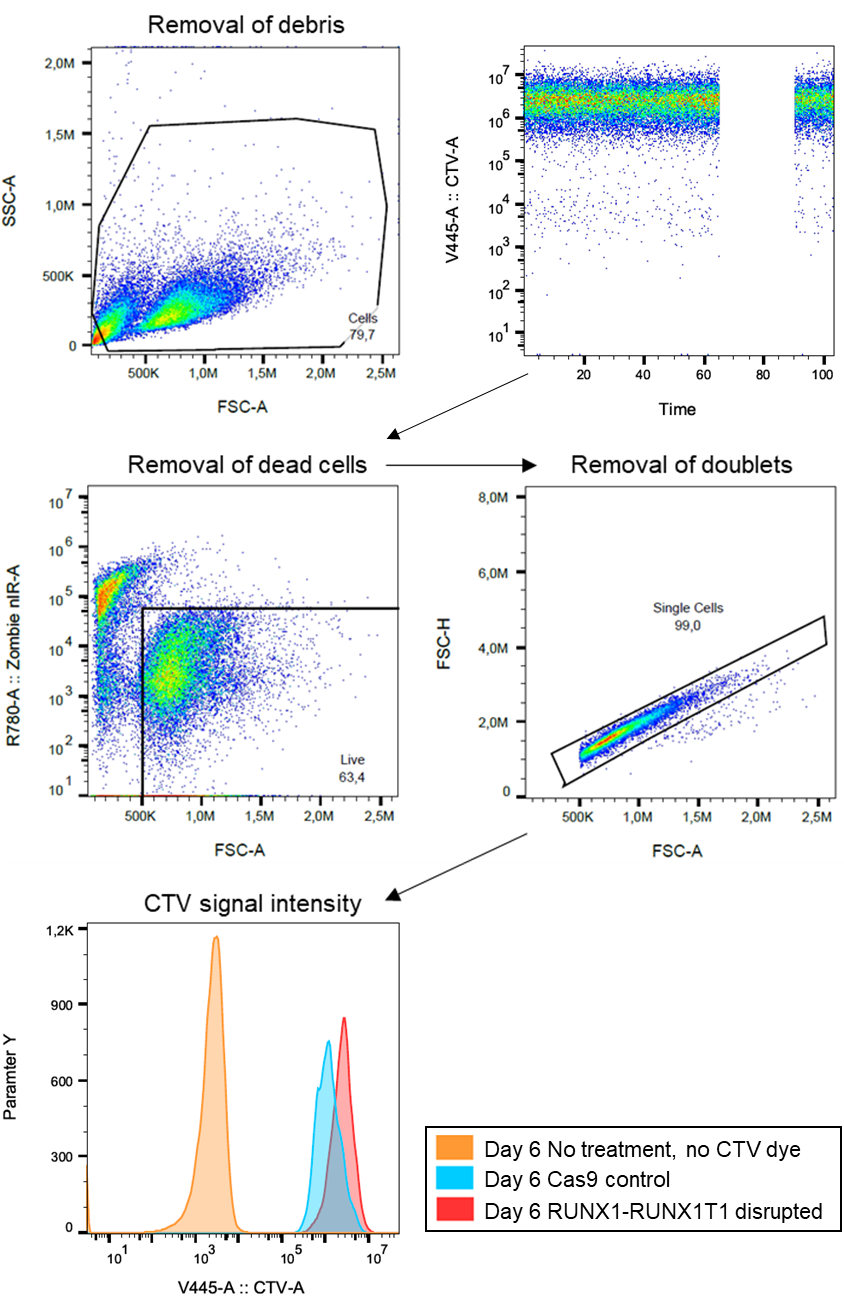
**

**Supplementary Figure 2**

Gating strategy for CRISPR-Cas9 treated THP-1 cell populations and Cas9 controls to obtain a measure of the cell trace violet (CTV) median fluorescence intensity (MFi) from live THP-1 cells on days 2, 4, 6, 9 and 11 following CRISPR-Cas9 treatment compared to controls. Here, gating is shown for THP-1 cells 6 days following CRISPR-Cas9 treatment. The same strategy was used on data from day 2, 4, 9 and 11. Zombie NIR was used as viability marker.

**
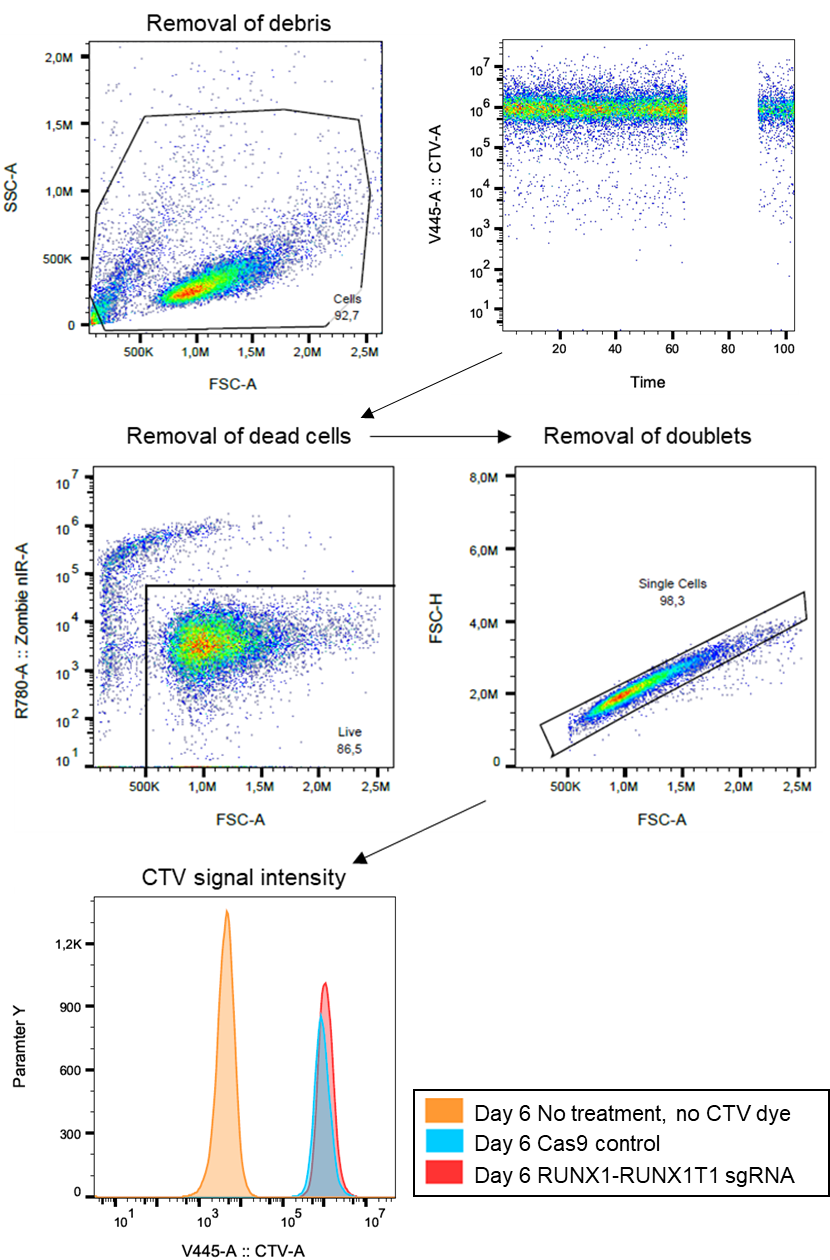
**

**Supplementary Figure 3**

*In silico* sorting of gated CTV high and CTV low Kasumi-1 subpopulations on data from ***Figure 4***. Sorting was based on MFi of Cell Trace Violet (CTV) dye on day 9 and 11 following CRISPR-Cas9 based *RUNX1-RUNX1T1* disruption. Here, histograms were produced by the gating strategy presented in ***Supplementary Figure 1*** on data from ***Figure 4***. The gates defining CTV high and CTV low populations were set by splitting the *RUNX1-RUNXT1* disrupted population on day 9 and 11 into two subpopulations at the CTV signal intensity peak valley.

**
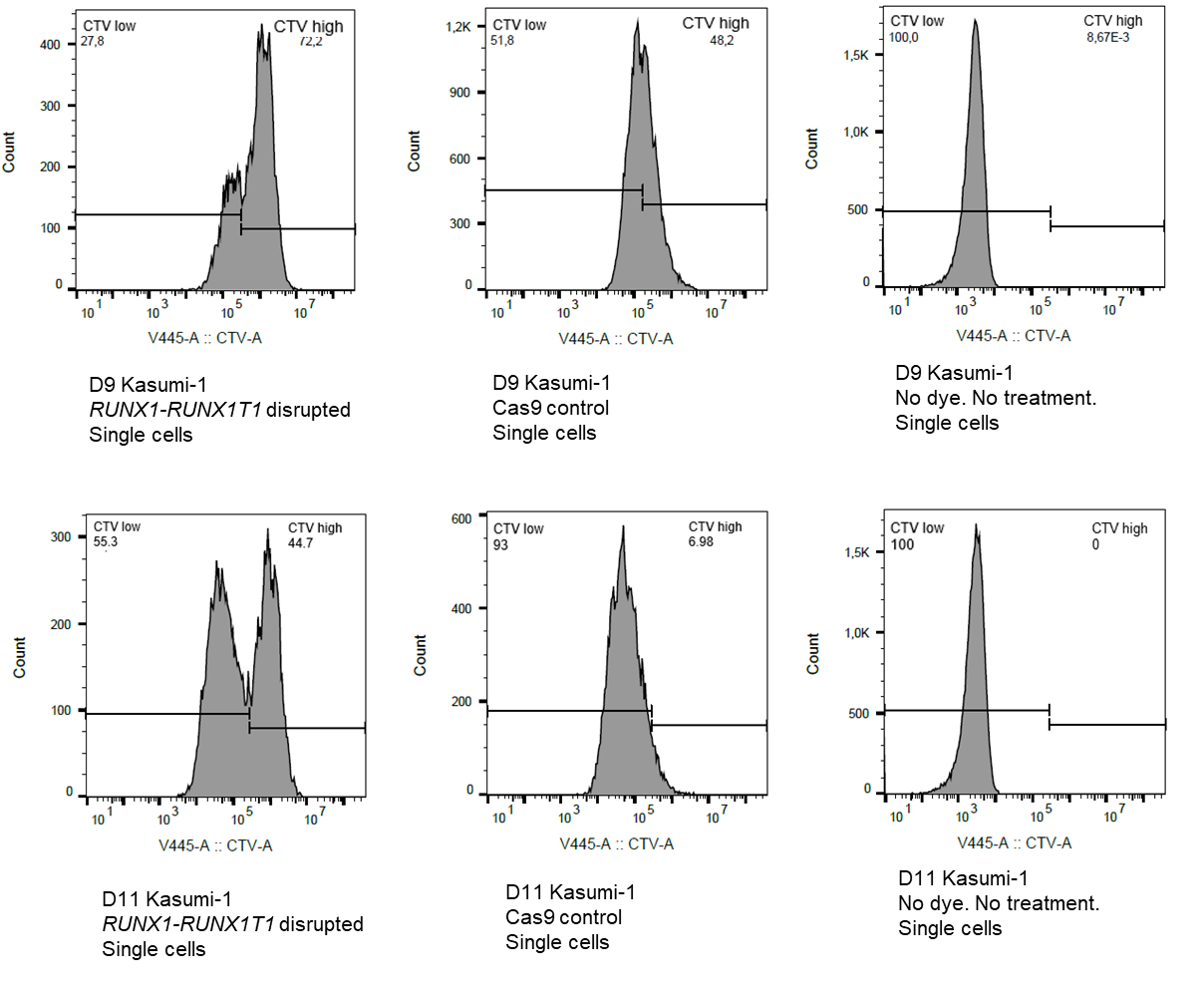
**

**Supplementary Figure 4**

Gating strategy for sorting of *RUNX1-RUNX1T1* disrupted Kasumi-1 cell population. Sorting was based Cell Trace Violet (CTV) dye MFi on day 11 following CRISPR-Cas9 disruption of *RUNX1-RUNX1T1*. PI was used as viability marker.

**
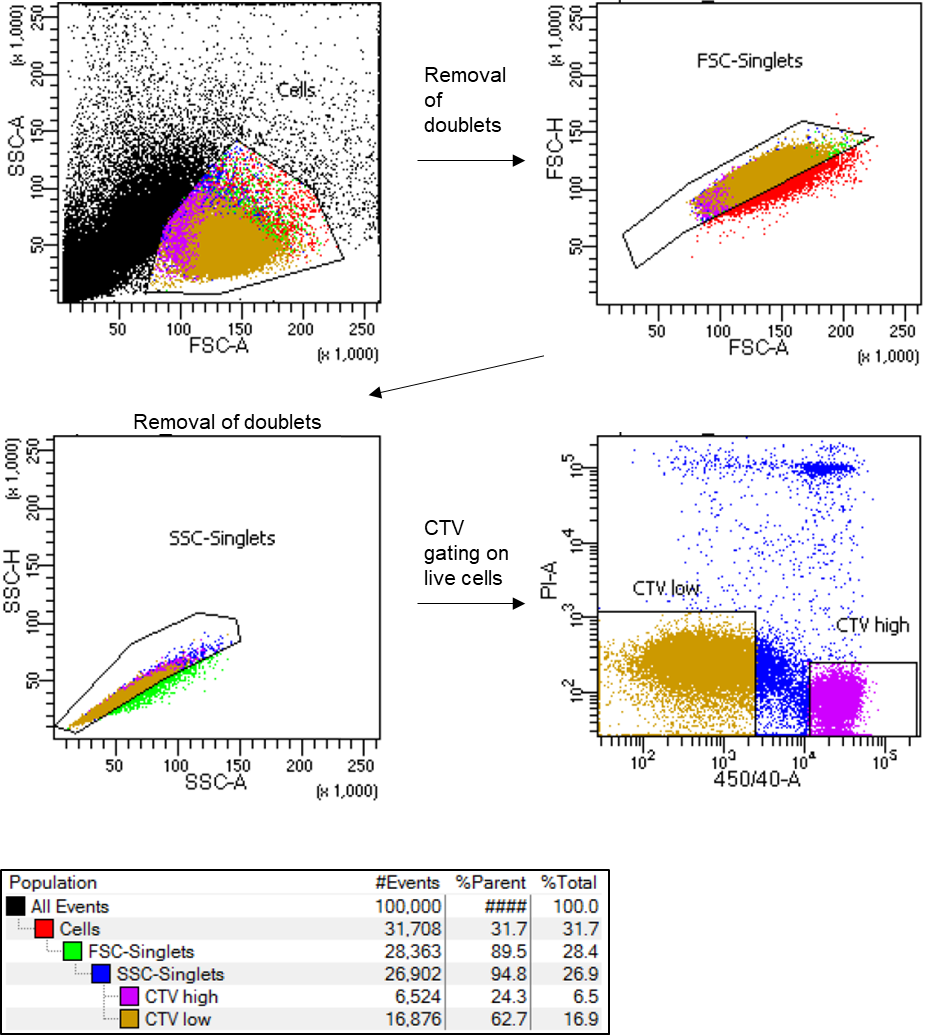
**

**Supplementary Figure 5**

**RUNX1-RUNX1T1 protein truncation following CRISPR-Cas9-mediated RUNX1-RUNX1T1 disruption**

Amino acid sequences of the native, edited and truncated RUNX1-RUNX1T1 protein, respectively. Residues corresponding to RUNX1 and RUNX1T1 are colored light and dark blue, respectively. Edited out residues are in strikethrough. New residues caused by frameshift are in red.


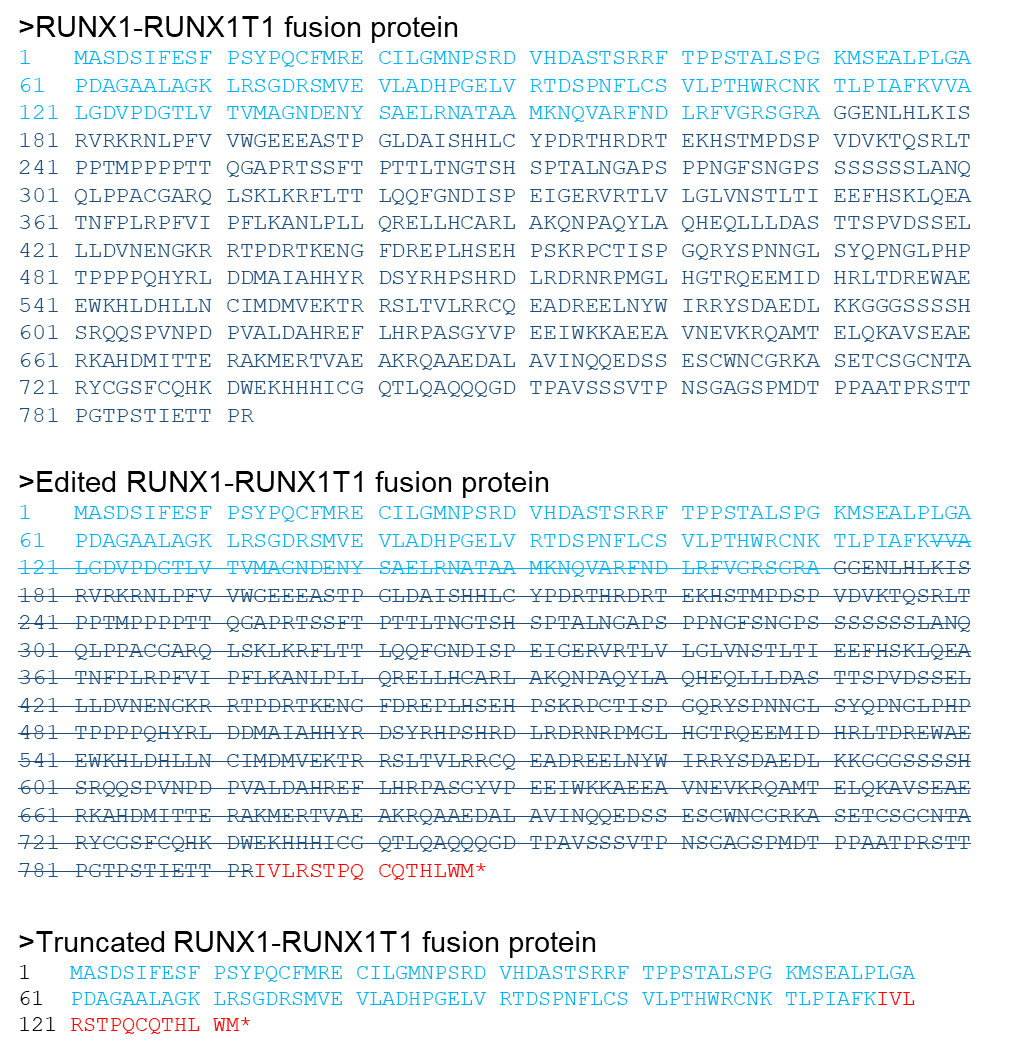


**Supplementary Figure 6**

48 off-target sites for RX2 sgRNA, predicted using CRISPRoff software, were sequenced for patient 1, patient 3 and cell lines THP-1, and Kasumi-1. Outliers at position chr7_116494795_116494827 are due to polymorphisms and not a result of off-target editing.


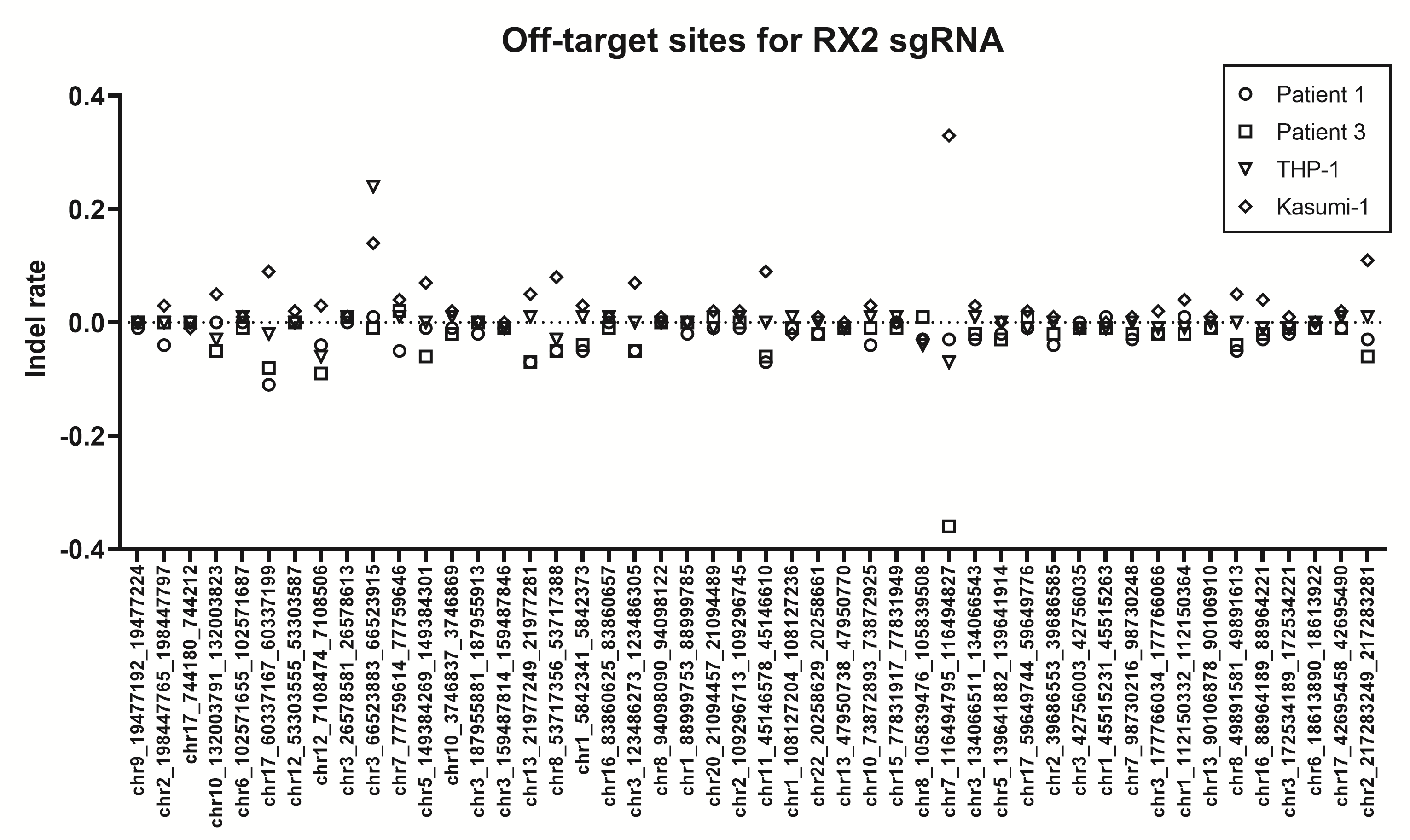


**Supplementary Figure 7**

Indel variants at chr7_116494795_116494827. Table on the right shows indel frequencies. The black box encompasses the predicted off-target site and PAM sequence. The vertical black line indicates the theoretical cleavage site of Sp-Cas9 at PAM +3. These indels are not caused by off-target activity, but are a result of polymorphisms as the same indel is present in both control and edited sample.

**
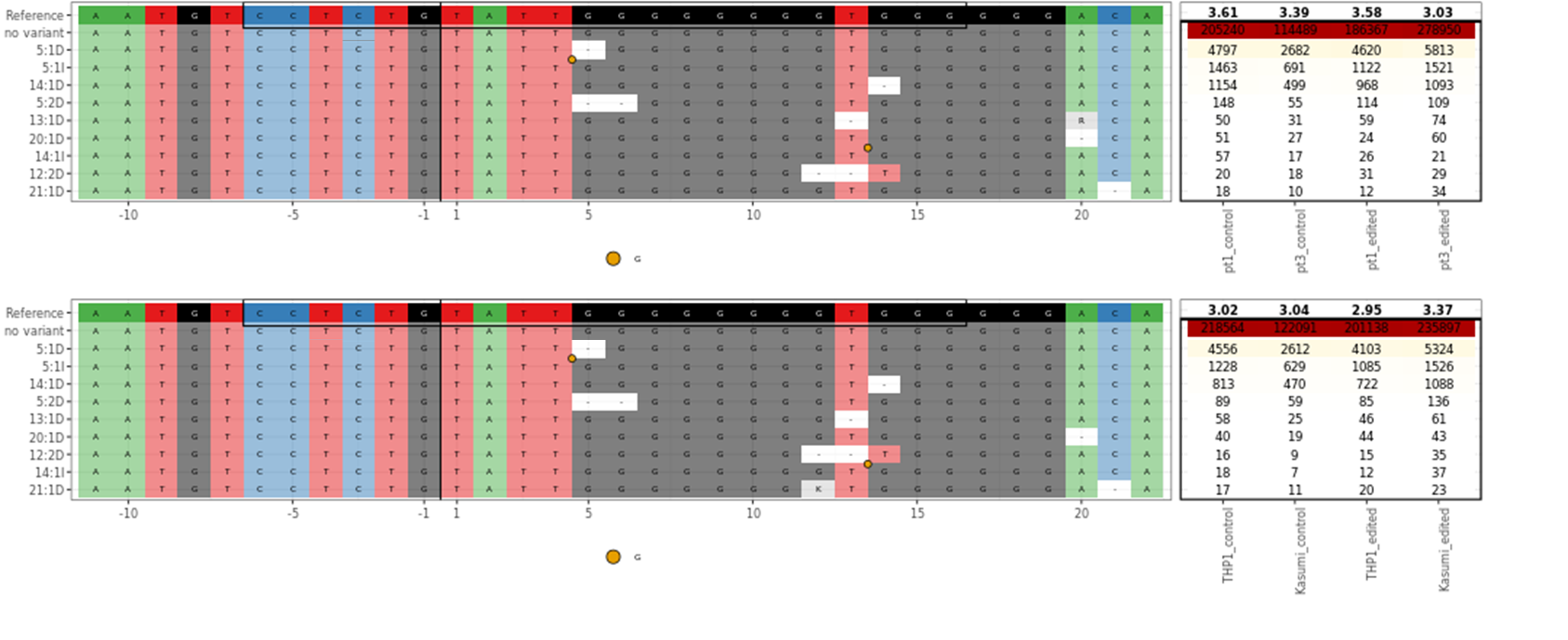
**

**Supplementary Figure 8**

42 off-target sites for RXT1 sgRNA, predicted using CRISPRoff software, were sequenced for patient 1, patient 3 and cell lines THP-1, and Kasumi-1. Outliers at positions chr6_13122973_13123005, chr16_89733731_89733763 and chr19_28132700_28132732 are due to polymorphisms and not a result of off-target editing.


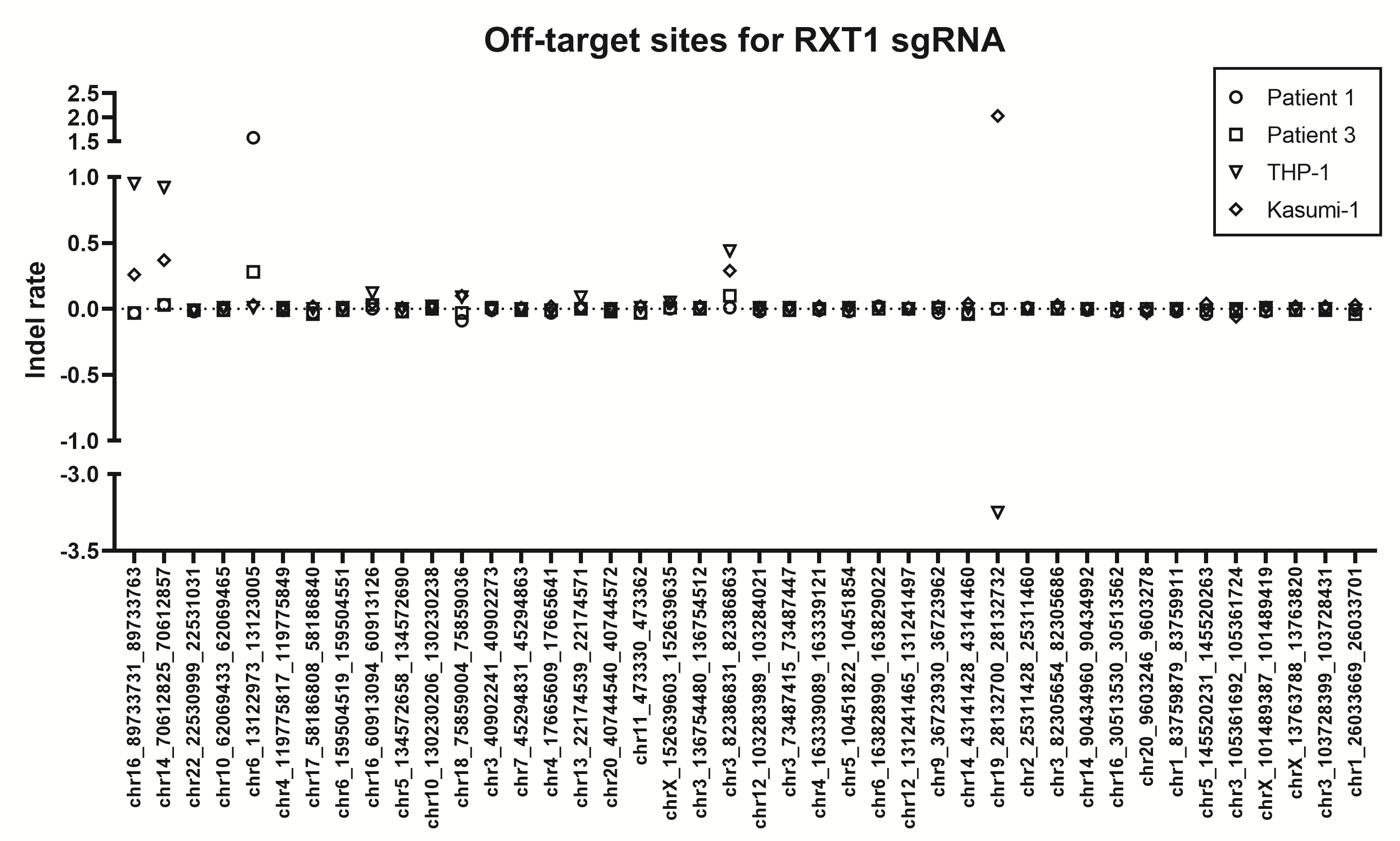


**Supplementary Figure 9**

Indel variants at chr6_13122973_13123005. Table on the right shows indel frequencies. The black box encompasses the predicted off-target site and PAM sequence. The vertical black line indicates the theoretical cleavage site of Sp-Cas9 at PAM +3. These indels are not caused by off-target activity, but are a result of polymorphisms as the same indel is present in both control and edited sample.


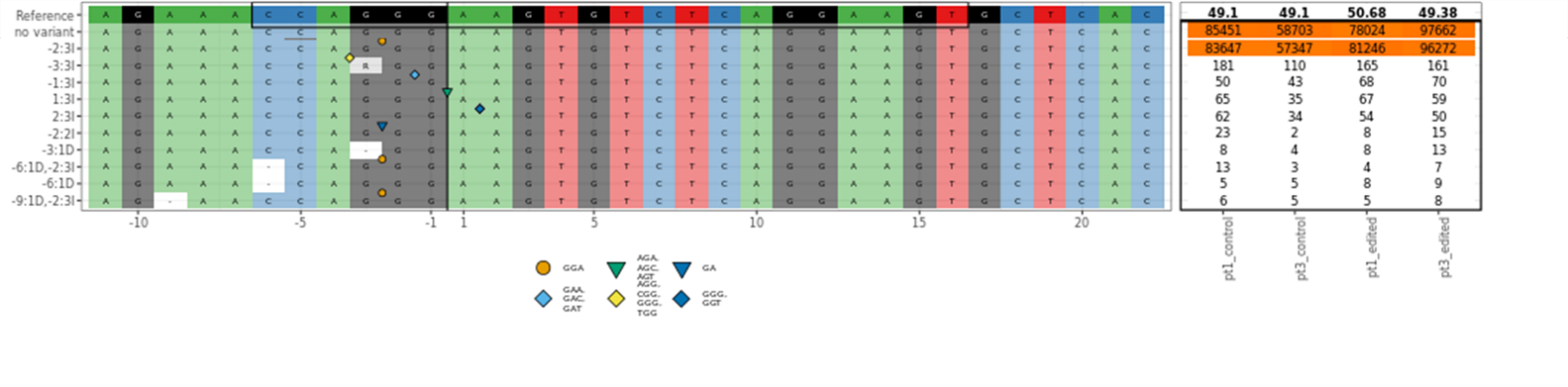
Indel variants at chr16_89733731_89733763. Table on the right shows indel frequencies. The black box encompasses the predicted off-target site and PAM sequence. The vertical black line indicates the theoretical cleavage site of Sp-Cas9 at PAM +3. These indels are not caused by off-target activity, but are a result of polymorphisms as the same indel is present in both control and edited sample.


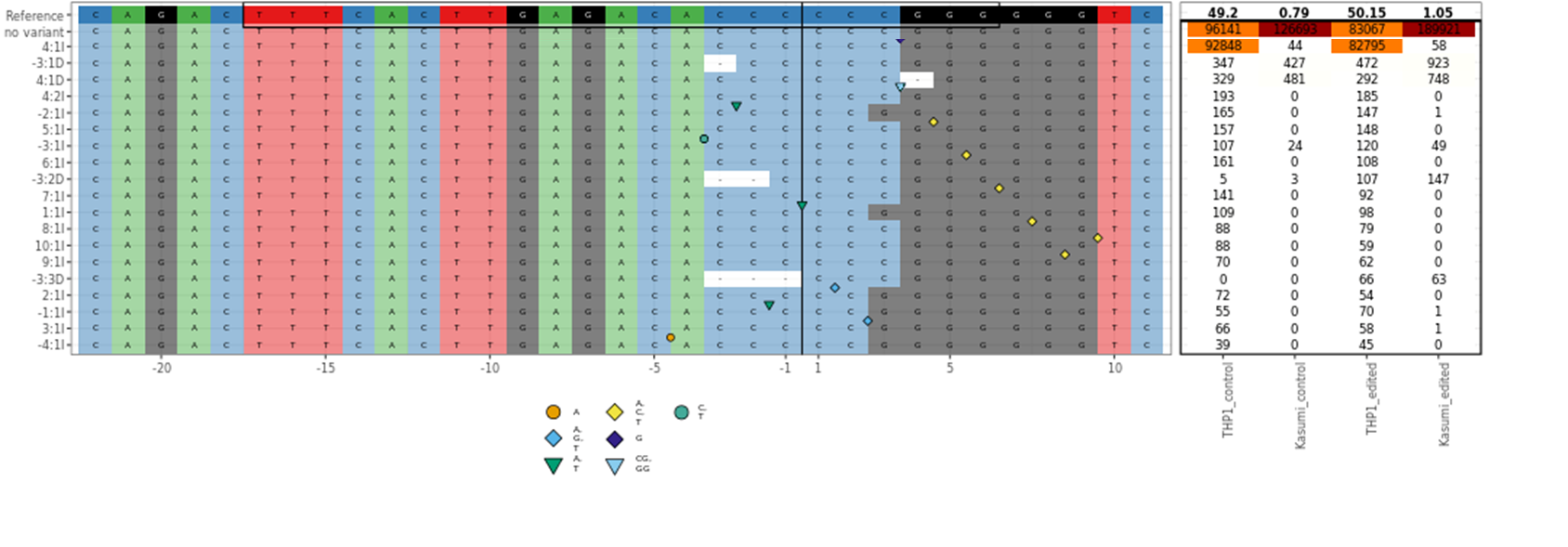
Indel variants at chr19_28132700_28132732. Table on the right shows indel frequencies. The black box encompasses the predicted off-target site and PAM sequence. The vertical black line indicates the theoretical cleavage site of Sp-Cas9 at PAM +3. These indels are not caused by off-target activity, but are a result of polymorphisms as the same indel is present in both control and edited sample.

**
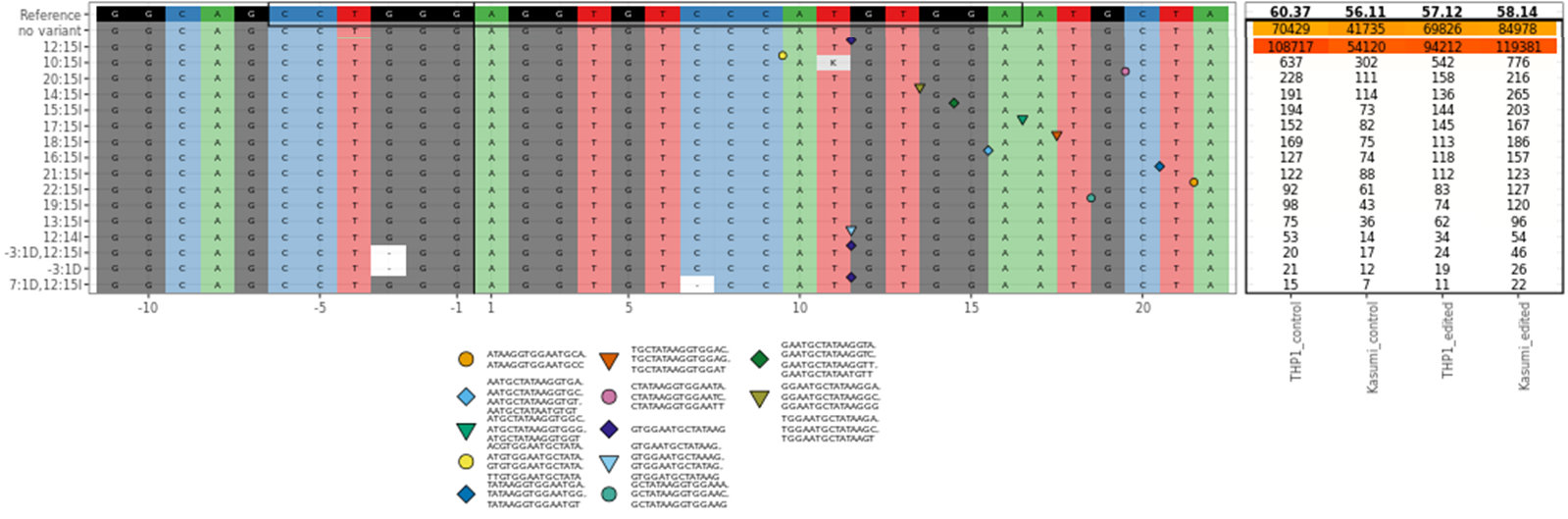
**

**Supplementary Figure 10**

**Formation of a disrupted chr. 8;21 translocation in THP-1 cells.**

1. PCR analysis of CRISPR-Cas9 cleavage products 3, 6 and 11 days following electroporation delivery of RNPs (RX2-RXT1) to Kasumi-1 (left) and THP-1 cells (right). Theoretical PCR product sizes (bp) for RX1-RXT2 = 391; no guides: 24 416. Green lines at 15 and 1 000 bp: capillary electrophoresis alignment marker.
2. Aligned Sanger sequencing data of RX2-RXT1-treated PCR products from Kasumi-1 and THP-1. RX2-RXT1 intron breakpoint sequence: CCCTGG.


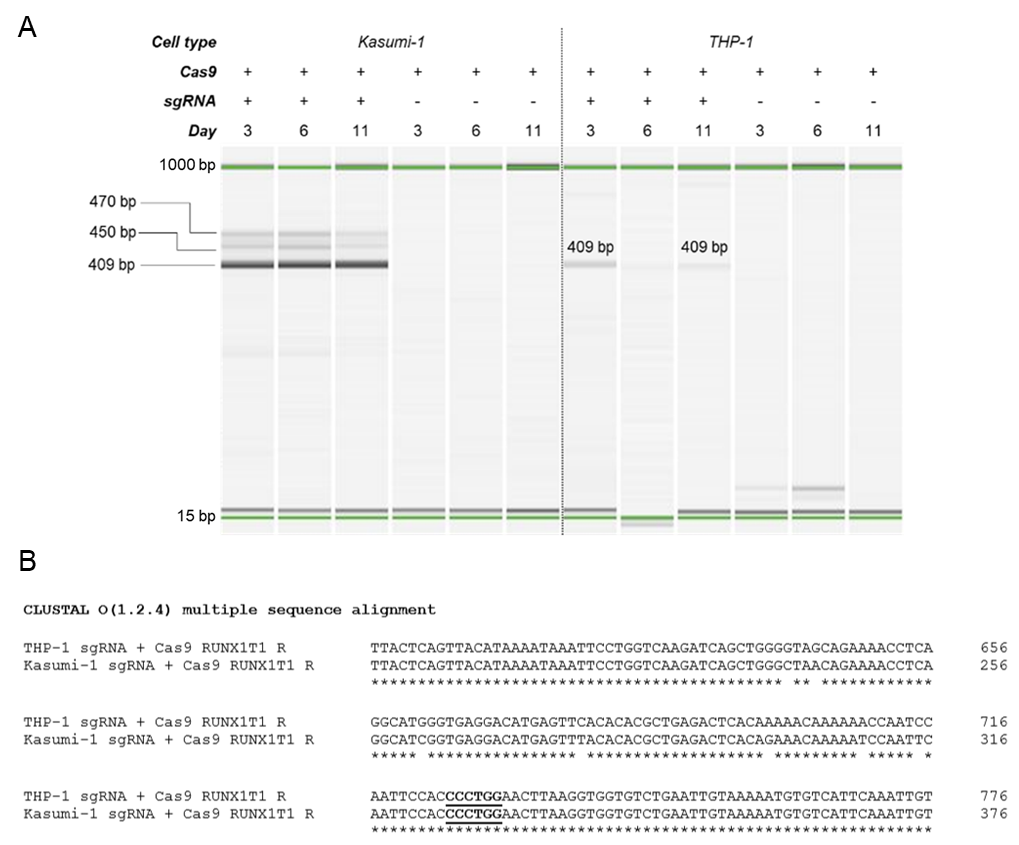


**Supplemental Figure 11**

**Persistence of chromosomal translocations in cell lines not harboring the *RUNX1-RUNX1T1* fusion gene.**
Cell lines THP-1 and MJ26146 were treated with sgRNAs RX2-RXT1 and Cas9 protein using electroporation for transfection. Following electroporation, cells were analyzed with PCR to detect the gene fusion between chromosomes 8 and 21, corresponding to the disrupted *RUNX1-RUNX1T1* as seen in Kasumi-1 cells. (A) MJ26146 were analyzed days 0, 3, 6 and 10. (B) THP-1 cells were analyzed days 0, 3 and 6.
Control *RUNX1* primers: *RUNX1 F* and *RUNX1 R*.
*RUNX1-RUNX1T1* fusion primers: *RUNX1 F* and *RUNX1T1 R*.
Theoretical PCR sizes (bp) *RUNX1 F* – *RUNX1 R*: 406; *RUNX1 F* – *RUNX1T1 R*: 391.


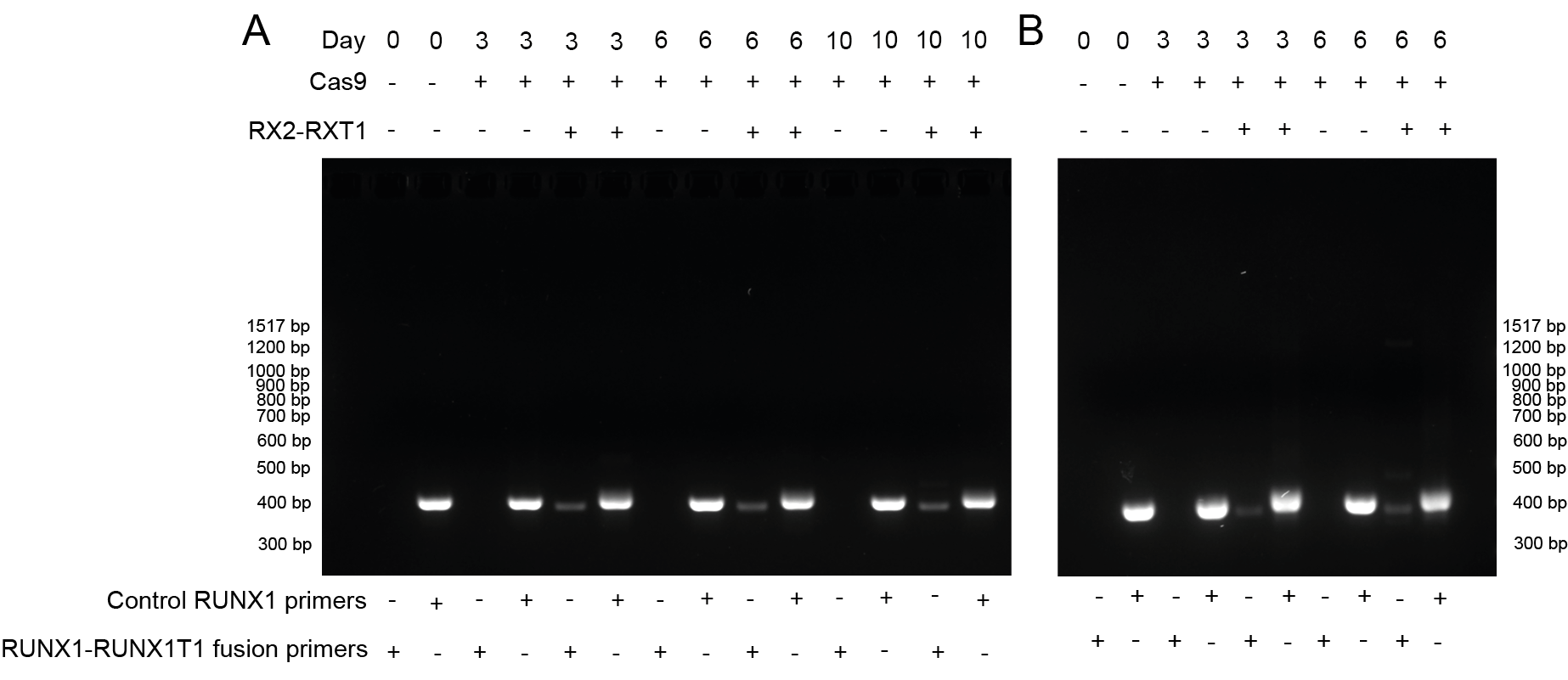


**Supplementary Figure 12**

Aligned Sanger sequencing data from RX1-RXT2-treated PCR products from an AML t(8;21) patient 4. RX1-RXT2 intron breakpoint sequence is underlined. Orange sequence: RX2 sgRNA. Green: RXT2 sgRNA.

**
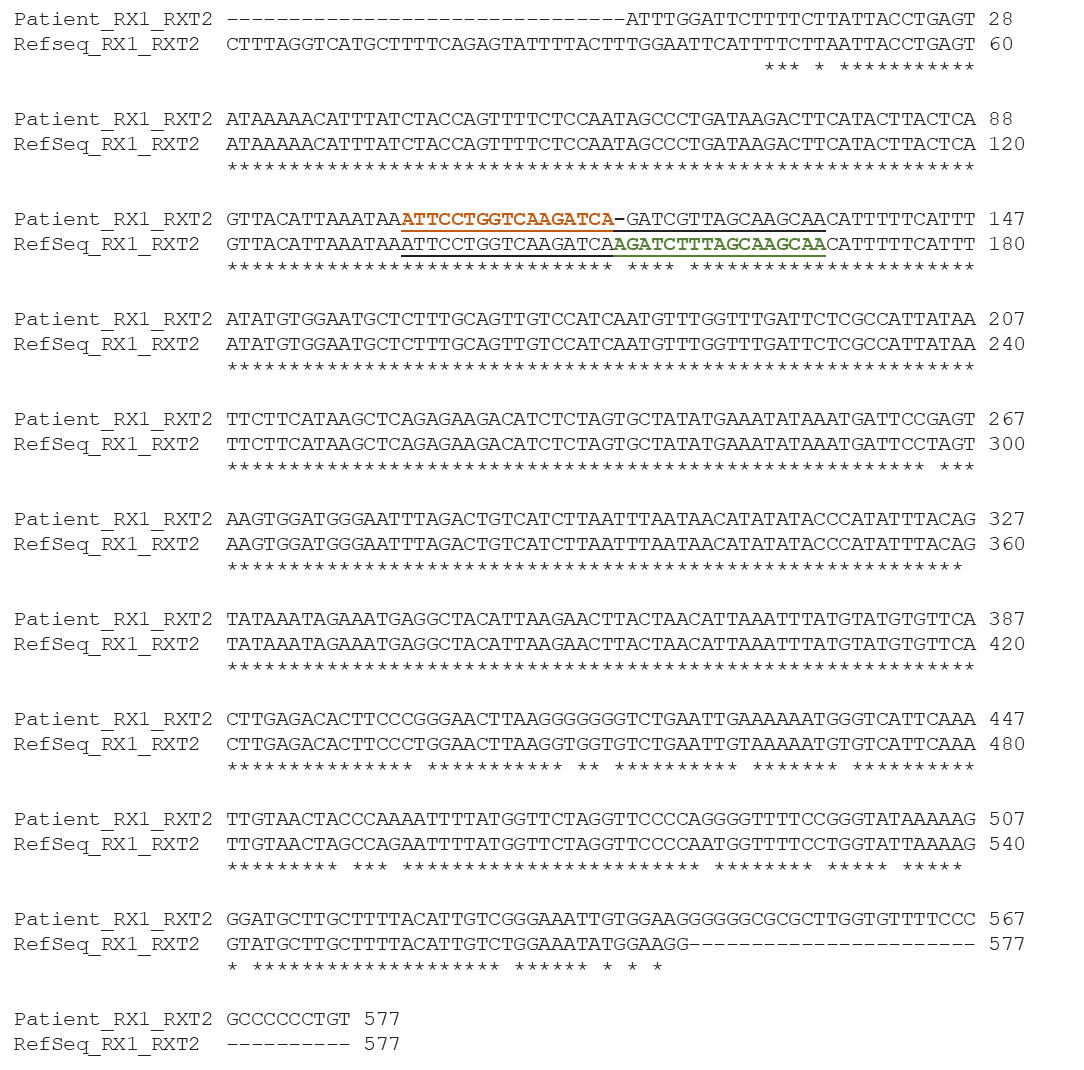
**
